# Mortality and Sickness in the City of Bristol for the Year 1903

**Published:** 1904-03

**Authors:** D. S. Davies

**Affiliations:** Medical Officer of Health, Bristol


### MORTALITY AND SICKNESS IN THE CITY OF BRISTOL FOR THE YEAR 1903.

BY

D. S. DAVIES, M.D. Lond.

*Medical Officer of Health, Bristol.*

---

THE general death rate for the year 1903, uncorrected for sex and age distribution, is 14.22 per 1000 living, the lowest rate yet recorded for the city. While this is satisfactory, it is more important to note that the average rate for the past five years has not exceeded 17.1, a point at or below which, as suggested by Farr, the causal influence of general insanitary conditions upon the death rate apparently ceases, lower rates indicating a response to abnormally favourable conditions, such as, for example, the continued absence of extremes of heat or of cold, with their respective fatal influence upon the very young and the aged.

The infant mortality rate, a delicate index to the sanitary balance of a population, is 116.3, the lowest recorded rate in the city. The infantile rate usually compares very favourably with that for the large towns of England; but the remarkable drop in 1903 is no doubt due to the continuous low temperature in the late summer and autumn, to the unusually mild winters, and to the absence of any fatal infantile complaint such as measles, which proved so fatal in 1902.

### THE PRINCIPAL EPIDEMIC DISEASES.

It may be of interest to indicate the actual mortality for five years (1898-1902) from seven diseases known for convenience as "zymotic."

| | |
|---|---|
| Diarrhœa ... ... ... ... | 1102 |
| Measles ... ... ... ... | 965 |
| Whooping Cough ... ... ... | 576 |
| Diphtheria ... ... ... ... | 493 |
| Enteric fever ... ... ... ... | 204 |
| Scarlet fever ... ... ... ... | 168 |
| Small-pox ... ... ... ... | 1 |

*Small-pox.*—During the year 1903 small-pox showed considerable activity, and in the first six months of the year was introduced into the city on thirteen occasions up to and including a case on June 4th. This corresponds to an introduction every fortnight. And these thirteen introductions, which affected practically every sub-district of the city, including Avonmouth (except St. George), and, owing to the extremely mild character of the disease, had generally become multiple before recognised and notified, resulted in 44 cases and 3 deaths. At the end of June the city remained free from any infection of small-pox. Seven of the thirteen introduced cases were reported early and were sterile of result. After June only two further introductions occurred, one from near Manchester, the other from Smyrna; no extension resulted.

Our local experience of small-pox is that it is the only disease (perhaps excepting typhus) in which one may count upon absolute success from the adoption of rational measures of control; a result partly of its straightforward habits, facilitating inhibition of its lines of spread, partly of its obligate parasitism and complete death upon exhaustion of soil, thus excluding "carrier" cases, and partly of its characteristic onset and eruption, recognisable to the initiated, in practically every case, however abnormally mild. No return case of small-pox has ever occurred within my experience.

*Diphtheria.*—This is an example of a far different disease. Probably always personally carried and spread; apparent recovery, though it indicates individual immunity, by no means excludes continued growth of the organism in some portion of the respiratory tract, with a high degree of potential danger to others. During the year 119 deaths have resulted from this disease, compared with 189 in 1902: there is, therefore, reason to believe that the invasion of the city by the virulent form of diphtheria, which began in 1900 after a long period of immunity extending over more than twenty years, is on the decline, and that succeeding years will show progressively satisfactory improvement.

*Scarlet Fever.*—The prevalence of this disease, though widespread, is also accompanied by a declining mortality; only 49 deaths resulted, compared with 66 in 1902. The epidemic wave which began to rise in 1900 has been maintained over a longer period than was usual before extension of the city in 1897, owing to the addition of 100,000 persons in districts largely composed of the working classes, yielding with their families much additional susceptible material to which the infection extends from the old city or *vice versâ:* there is now, in fact, a double city to reckon with, and consequently a prolonged period of epidemic prevalence.

*Enteric Fever.*—The number of deaths from this disease in 1903 was 21, corresponding to a lower rate than has occurred for the past twelve years. As an index of general sanitary condition this rate is of some importance.

*Phthisis.*—The number of deaths from pulmonary consumption registered during the year was 466, while the seven principal epidemic diseases caused only 375 deaths in all. The Council has resolved to adopt during 1904 a system of voluntary notification of phthisis, so that useful advice may be distributed where most needed, and disinfection of premises and infected articles secured. Twenty beds have been secured at the Winsley Sanatorium for the isolation and treatment of suitable cases from the city not belonging to the pauper classes.

A bye-law against spitting in public rooms and conveyances has also been adopted.

*Measles.*—Only 11 deaths were reported in 1903 from this disease, which caused 411 in 1902.

*Whooping Cough* caused 65 deaths in 1903, compared with 115 in 1902.

*Puerperal Fever.*—Fourteen deaths are returned from this cause, a smaller number than in any of the past four years.

### The Midwives Act, 1902.

This Act came into force on April 1st, 1902, and is administered by the City Council, who have delegated the powers and duties under the Act to the Watch Committee.

Under this Act it becomes a punishable offence:—

1. After the 1st of April, 1905, for any woman not certified under the Act to take or use the name or title of Midwife, or any name, title or description implying that she is certified or is recognised by law as a midwife.

2. After the 1st of April, 1910, for any woman not certified under the Act to practice as a midwife otherwise than under the direction of a qualified medical practitioner.

The following provision is made for existing midwives.

Any woman who, within two years from the date of the Act coming into operation, claims to be certified under the Act, shall be so certified provided she holds a certificate in midwifery from the Royal College of Physicians of Ireland, or from the Obstetrical Society of London, or the Coombe Lying-in Hospital and Guinness's Dispensary, or the Rotunda Hospital for the Relief of the Poor Lying-in Women of Dublin, or such other certificate as may be approved by the Central Midwives Board, or produces evidence, satisfactory to the Board, that at the passing of this Act she had been for at least one year in *bonâ-fide* practice as a midwife, and that she bears a good character.

Medical practitioners might advise midwives as to the provisions of the Act, and applications for Registration should be made to the Town Clerk.

---

